# The Impact of Cold Storage and Seasonality on Raw Cow Milk Microbiota, Assessed by Conventional and Viability PCR

**DOI:** 10.3390/ani16152302

**Published:** 2026-07-24

**Authors:** Phong Dinh Tran, Takeshi Tsuruta, Naoki Nishino

**Affiliations:** 1 Graduate School of Environmental, Life, Natural Science and Technology, Okayama University, Okayama 700-8530, Japan; dinhdinh0913@gmail.com (P.D.T.); tsurutafe@okayama-u.ac.jp (T.T.); 2 Research Center for Intestinal Health Science, Okayama University, Okayama 700-8530, Japan

**Keywords:** cow milk, cold storage, microbiota, seasonal variation, viability

## Abstract

The microbiota of healthy Holstein cow’s milk was analyzed to evaluate the effects of immediate (freezing at the farm: on-site workflow) and delayed processing (freezing after cool transport: laboratory workflow) and to investigate seasonal changes in September, November, and January. Propidium monoazide (PMA) was also used to distinguish between viable and non-viable cells. Microbiota composition differed between the laboratory and the on-site workflow. After cool transport, the abundances of *Lactobacillus* and *Turicibacter* increased, while those of *Streptococcus*, *Bradyrhizobium*, and *Acinetobacter* decreased. Based on the β-diversity assessment, the difference between viable and non-viable cells was marginal compared with the difference between on-site and laboratory workflows. In the subsequent on-site workflow experiment, seasonal variation was clearly demonstrated. The relative abundances of *Lactobacillus*, *Turicibacter*, and *Bacillus* were high in September; *Staphylococcus*, *Phenylobacterium*, and *Bradyrhizobium* in November; and *Phyllobacterium* in January. The seasonal effect was greater than the PMA treatment effect. Despite the study’s limitations, such as a limited number of milk samples and data from only one farm with a single management system, our findings indicate that storage and transport at low temperatures could lead to inaccurate assessments, particularly of opportunistic environmental microbiota. Viability PCR could help improve our understanding of the factors involved but would not substantially alter the raw milk microbiota.

## Simple Summary

Raw milk microbiota collected from the udders of non-mastitic Holstein cows was assessed differently between samples immediately frozen on-site and those transported to the laboratory under cool conditions. The abundances of opportunistic environmental taxa, i.e., *Bradyrhizobium*, *Ralstonia*, *Acinetobacter*, and f_Sinobacteriaceae, decreased, whereas those of milk processing- and gut-associated taxa, i.e., *Lactobacillus*, *Turicibacter*, and f_Clostridiaceae, increased. Typical mastitis-related taxa, including *Streptococcus*, *Staphylococcus*, *Corynebacterium*, f_Enterobacteriaceae, and *Mycoplasma*, as well as psychrotrophic *Pseudomonas* and *Acinetobacter*, were largely unaffected. Viability PCR using propidium monoazide further altered the raw milk microbiota, but the difference between viable and non-viable cells was marginal compared with the difference observed due to delayed processing. Seasonal variation of raw milk microbiota, which has been demonstrated by many studies, was clearly evident in assessments based on immediately processed samples. These findings indicate that sample handling from collection onward can support reliable interpretation of udder milk microbiota for dairy cow health and product quality management.

## 1. Introduction

A diverse range of bacteria is detected in raw milk; their composition varies with herd management, environment and hygiene, including lactation stage, udder health, climate, feed, bedding, and milking systems [1,2,3]. Since these variables are closely linked to the profitability and sustainability of milk production [1,4], understanding the raw milk microbiota and exploring control methods are of great importance. Advances in culture-independent analyses and associated bioinformatics have helped gain insights into the dynamics of potential pathogens and opportunistic non-pathogens [5,6]. Although predicting and preventing mastitis through analysis of the raw milk microbiota remains challenging, monitoring the microbiota is considered vital for improving the management of dairy cow health and milk product quality [7,8].

Milk samples collected from individual quarters [9], bulk tanks [2], and large silos [10] have been examined depending on the objective. Although analyzing the microbiota of udder milk is required to understand the health status of individual cows [9,11], analyzing storage tank milk, which enables large-scale surveys across numerous farms and factories, can provide up-to-date information to the whole industry and dairy operations [2,10,12]. Interestingly, both udder milk and bulk tank milk surveys have reported seasonal variations [3,10,12,13,14,15], suggesting that the raw milk microbiota of healthy cows can be influenced by environmental factors at the herd level rather than at the individual cow level [1,2,16]. This contrasts with the observation that microbiota in human breast milk may remain stable throughout the year [17,18], thereby indicating that repeated milking with strong mechanical pressure causes substantial damage to cows’ udders. In assessing raw milk microbiota, sample storage and processing workflows may also influence the results [5,19,20,21]. Psychrotrophic bacteria, such as *Pseudomonas* and *Acinetobacter*, can be enriched during cold storage [4,20,21]; consequently, the prevalence of thermophilic and mesophilic bacteria may be underestimated in bulk-tank milk compared with udder milk samples [4,21]. Representative mastitis-causing pathogens, such as *Staphylococcus*, *Streptococcus*, and *Escherichia*, are not psychotropic [4,9]; hence, if their relative abundance decreases due to the enrichment of psychrotrophs, the mastitis pathogens may be underestimated. Reports suggest that psychrotrophic Actinobacteria warrant attention as spoilage organisms due to their proteolytic and lipolytic activities [21]; hence, the prevalence of psychrotrophic bacteria needs to be correctly estimated.

Given that cold storage affects microbiota composition, the handling procedure for raw milk samples between farm collection and laboratory analysis, as well as differences between individual and bulk-tank milk, may influence the resulting data [1,21]. Most of the published studies examined milk samples stored at cool temperatures for hours, from the farm to the laboratory, regardless of whether the samples were from udders or bulk-tank [1,5]. Person et al. [22] found no marked differences in microbiota of a wild animal (California ground squirrels) between fecal samples frozen immediately in liquid nitrogen on-site and those frozen after several hours of storage on ice, whereas Kamilari et al. [23] reported substantial changes in the microbiota of goat milk between samples frozen immediately after sampling and after the overnight cold storage. The taxa and populations of microbiota inhabiting feces and cow milk differ substantially; hence, the effects of cold storage may also differ and warrant further study.

Discriminating viable and non-viable cells could refine our understanding of the relationship between raw milk microbiota, cow health, and product quality [24,25,26]. Although culture-independent analyses have clarified the roles and functions of microbiota that are difficult to characterize with conventional culture-based techniques, they do not distinguish viable from non-viable cells, provided that the DNA sequences used as taxonomic markers remain amplifiable [26,27,28]. For bacteria enriched during cold storage, the proportion of viable cells may increase, whereas for those depleted, the proportion may decrease [24,26]. Apart from considering the effects of cold storage, it remains unclear which bacterial taxa detected in raw milk are actually viable, and whether certain taxa are more likely to be detected as viable or non-viable cells [24,25,26]. Excluding dead cells with propidium monoazide (PMA) has been shown to reduce alpha diversity and alter the relative abundance of the rumen [29] and saliva [30] microbiota. Likewise, two-thirds of the human breast milk microbiota were shown to originate from non-viable cells [28]. In contrast, Kable et al. [24] found no advantage to using viability PCR when analyzing the microbiota of cold-stored silo milk until the milk was heat-pasteurized. How sample storage and processing workflows should influence the resulting microbiota of raw cow’s milk, and whether seasonal variation can be evaluated differently when viability PCR is applied, warrant examination.

The objective of this study was to describe appropriate methods for monitoring the raw milk microbiota to support improved management of dairy cow health and milk product quality. In Experiment 1, milk samples were collected from the four quarters of healthy lactating Holstein cows in late autumn (November 2023), and two handling procedures, i.e., immediate on-site freezing and freezing after several hours of cold storage on ice, were compared, with and without PMA treatment. In Experiment 2, milk samples collected from the four quarters in late summer (September 2023) and mid-winter (January 2024) were immediately frozen on-site and included in the analyses. Seasonal variations in the raw milk microbiota were examined, including late autumn data, and the viability PCR, which discriminates viable from non-viable cells, was validated.

## 2. Materials and Methods

### 2.1. Sample Collection

Milk samples were collected from 13 lactating Holstein cows at the Chugoku-Shikoku Dairy College. The collection of milk samples began in September 2023 and continued in November 2023 and January 2024. At the commencement of sampling in September 2023, mean ± standard deviation for the parity and days in milk of the cows were 2.2 ± 1.4 and 144 ± 80.1, respectively. Several cows were replaced during the sampling period. More samples were collected from multiparous cows, with a primiparous-to-multiparous ratio of about 4:6. The cows were milked twice daily at 06:00 and 18:00 using a pipeline milking system, and morning milk was collected. The cows were housed in tie-stalls and fed a diet formulated with a forage-to-concentrate ratio of 50:50. Based on milk yield, the concentrate proportion was adjusted individually. The forages consisted of grass silage, whole crop corn silage, and sudan grass hay. The first few streams of foremilk were discarded after cleaning the surface of the four teats. Subsequent streams from the four teats were collected in a 50 mL collection tube. All procedures and protocols were approved by the Animal Care and Use Committee of Okayama University, Japan (OKU-2022093).

### 2.2. Comparison Between Immediate and Laboratory Workflows (Experiment 1)

This study was conducted in November 2023. Each cow’s milk (*n* = 13) was divided into matched aliquots assigned to on-site and laboratory workflows. Within each workflow, samples were further divided into non-PMA control and PMA-treated samples. In the on-site workflow, samples were transported to the college building and then either frozen immediately in liquid nitrogen or frozen after PMA treatment, as described below. In the laboratory workflow, samples were transported on ice to the laboratory at Okayama University and then frozen in a freezer with or without PMA treatment. The delay was approximately eight hours. All samples were kept frozen at −30 °C until being processed for microbiota analyses.

### 2.3. Comparison Between Seasons (Experiment 2)

The sampling in the on-site workflow was conducted in September 2023 (*n* = 12) and January 2024 (*n* = 11). Each sample was divided into non-PMA control and PMA-treated samples. Seasonal variation was assessed using a total of 72 samples, including non-PMA control and PMA-treated November samples. The weather and minimum/maximum temperatures on the sampling days were as follows: rain and 17.5–26.3 °C in September; cloudy and −0.5–6.9 °C in November; and snow and −4.6–0.2 °C. September, November, and January are described as late summer, late autumn, and mid-winter in this study.

### 2.4. Propidium Monoazide Treatment

The PMA treatment was performed in the college building or in the university laboratory. To discard the fat and supernatant layers, 1.25 mL of milk sample was centrifuged for 5 min at 13,000 rpm. The resulting pellets were washed twice with 1 mL of phosphate-buffered saline, with each wash followed by centrifugation for 5 min at 13,000 rpm. Following the final wash, the pellets were resuspended in 500 µL of phosphate-buffered saline. For viability differentiation, 6.25 µL of PMAxx^TM^ dye (20 mM in H_2_O, Biotium, Fremont, CA, USA) was added to achieve a final concentration of 50 µM. The tubes were encased in aluminum foil and transferred to a dark drawer for a 15 min incubation, during which gentle mixing was performed every 5 min [25,26]. Following dark incubation, the foil was removed, and the tubes were positioned on a chilled ice surface. Light activation was achieved by exposing the samples to a 500 W halogen lamp for 15 min, with inversions conducted every 5 min to maintain homogeneity. The PMA-free pellets were suspended in 500 µL of plain phosphate-buffered saline and subjected to identical incubation and light exposure conditions as the treatment group. Final aliquots were then used for DNA extraction at the Animal Nutrition Laboratory, Okayama University.

### 2.5. DNA Extraction and Purification

Bacterial DNA was isolated using the repeated bead beating plus column protocol [31]. Initially, 0.4 g of zirconia beads, consisting of 0.3 g of 0.1 mm beads and 0.1 g of 0.5 mm beads, were placed into 2 mL screw cap tubes. Following the transfer of the total sample volume, 1 mL of lysis buffer, 500 mM NaCl, 50 mM Tris HCl at pH 8.0, 50 mM EDTA, and 4% sodium dodecyl sulfate, was introduced into each tube. The mixture was incubated at 70 °C for 15 min. Mechanical lysis was subsequently executed using a bead beater homogenizer (Beads Crusher μT-12, TAITEC, Saitama, Japan) at maximum speed for 3 min.

After centrifugation at 16,000× *g* at 4 °C for 5 min, the supernatant was collected, and the homogenization process was repeated with 300 µL of lysis buffer. Ammonium acetate was then added to a volume equivalent to one-third of the collected supernatant, followed by a 5 min incubation on ice and a 10 min centrifugation. The resulting supernatant was mixed with an equal volume of isopropanol and subjected to a further 30-min incubation on ice. Following 15 min of centrifugation, the supernatant was discarded, and the nucleic acid pellet was washed with 70% ethanol and dried under vacuum for 3 min. The pellet was resuspended in 200 µL of Tris EDTA at pH 8.0, with further purification conducted using the DNeasy Blood and Tissue Kit (Qiagen, Germantown, MD, USA) in accordance with the manufacturer’s instructions.

### 2.6. Quantitative PCR

To quantify the total bacterial population, the V3–V4 region of the bacterial 16S rRNA genes was amplified using the primer set (forward: 5′-TCCTACGGGAGGCAGCAGT-3′; reverse: 5′-GGACTACCAGGGTATCTAATCCTGTT-3′) [32]. For each 1 µL DNA sample, a 9 µL reaction premix was prepared consisting of 0.05 µL of each forward and reverse 10 µM concentrated primer, 5 µL of Brilliant III Ultra Fast SYBR Green QPCR Master Mix (Agilent Technologies, Santa Clara, CA, USA), and 3.9 µL of distilled water. After the premix was combined with either samples or standards, the 96-well plates were centrifuged and loaded into an AriaMx Real-Time PCR System (Agilent Technologies, Santa Clara, CA, USA). The qPCR thermal cycling conditions initiated with a denaturation step at 95 °C for 3 min, followed by 40 cycles of denaturation at 95 °C for 15 s, primer annealing at 60 °C for 1 min, and extension at 72 °C for 1 min. Fluorescent data were collected during a final melting curve analysis comprising steps at 95 °C for 30 s, 65 °C for 30 s, and 95 °C for 30 s. A standard curve ranging from 10^2^ to 10^7^ copies was generated using plasmid DNA prepared with 16S rRNA genes of *Escherichia coli*.

### 2.7. Amplicon Library Preparation and Sequencing

Using a two-step PCR process, the bacterial DNA from non-PMA control and PMA-treated samples was converted into amplicon libraries for next-generation sequencing. Primers targeting the V4 region of the 16S rRNA gene (forward: 5′-ACACTCTTTCCCTACACGACGCTCTTCCGATCTGTGCCAGCMGCCGCGGTAA-3′; reverse: 5′-GTGACTGGAGTTCAGACGTGTGCTCTTCCGATCTGGACTACHVGGGTWTCTAAT-3′) were used in the first round of PCR [33]. The PCR thermal cycling started with a denaturation phase of 94 °C for 2 min, followed by 35 cycles of 94 °C for 30 s, 50 °C for 30 s, and 72 °C for 30 s, and ended with a final elongation step of 72 °C for 5 min. The PCR products were purified using the Fast Gene Gel/PCR Extraction Kit (Nippon Genetics Co., Ltd., Tokyo, Japan), and purified first-round products served as templates for the second-round PCR with adapter-attached primers. Thermal cycling conditions consisted of an initial denaturation at 94 °C for 2 min, followed by 10 cycles of 94 °C for 30 s, 59 °C for 30 s, and 72 °C for 30 s, and a final elongation at 72 °C for 5 min. The purified second-round amplicons were subjected to 2 × 250 bp paired-end sequencing on an Illumina MiSeq platform at FASMAC Co., Ltd. (Kanagawa, Japan). For BioProject accession PRJDB42780, all sequencing data were uploaded to the NCBI Sequence Read Archive upon receipt as FASTQ files [34].

### 2.8. Bioinformatics

Microbiome data were processed using QIIME 2, version 2024.5, following the framework described for reproducible microbiome data science [35]. Raw reads were demultiplexed via q2-demux and denoised using DADA2 v1.30.0 [36], with forward and reverse reads truncated at 217 bp and 200 bp, respectively. After consensus chimera removal, unique amplicon sequence variants were aligned with MAFFT v7.520 [37] and used to construct a midpoint-rooted phylogenetic tree with FastTree v2.1.11 [38]. To normalize sequencing depth, samples were rarefied to a depth of 15,272 sequences, based on the minimum sample frequency [39]. Alpha diversity was evaluated using Chao 1 richness [40] and the Shannon diversity index [41]. Taxonomic assignment utilized a Naïve Bayes classifier trained on the Greengenes 13_8 database for the V4 region [42]. Bacterial clustering was analyzed at the phylum and genus levels.

### 2.9. Statistical Analysis

Statistical analyses were performed using JMP (version 14; SAS Institute, Tokyo, Japan), GraphPad (Prism 10, Boston, MA, USA), and Primer version 7 with Permanova+ add-on (Primer-E, Plymouth Marine Laboratory, Plymouth, UK). The richness and diversity of the milk microbiota were estimated using the Chao 1 and Shannon indices. Differences in relative abundance between groups were assessed using the nonparametric Wilcoxon test, and probability values less than 0.05 were considered significant. Microbiota data were visualized in R (version 4.3.3, R Foundation for Statistical Computing, Vienna, Austria) using the ggplot2 package (version 4.0.3) within the Rstudio (version 2025.09.1+401) integrated development environment. Differences in microbiota community structure were visualized using principal coordinates analysis (PCoA). Permutational multivariate ANOVA (PERMANOVA) was performed to assess the significance of workflow, season, and PMA treatment. Discriminant vectors with Pearson correlations > 0.7 were used to represent the taxa that accounted for the differences.

## 3. Results

### 3.1. On-Site and Laboratory Workflows

From the 52 samples, 2,082,152 high-quality reads were obtained, with per-sample counts ranging from 28,677 to 86,147, in Experiment 1. Read depth and non-chimeric counts were not affected by the on-site and laboratory workflows or by the PMA treatment.

**Figure 1:**
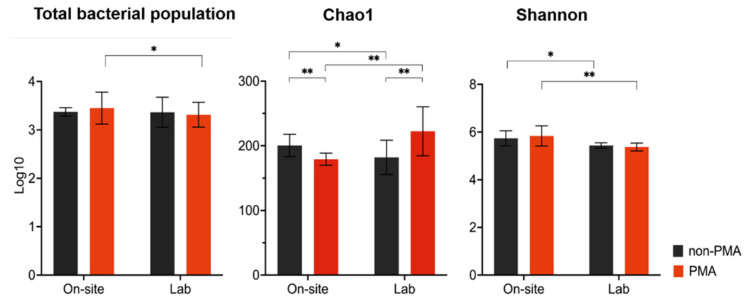
Total bacterial counts and alpha diversity indices in the bacterial microbiota of healthy cow’s milk subjected to immediate (freezing at the farm: on-site workflow) and delayed processing (freezing after cool transport: laboratory workflow). Viable and non-viable cells were discriminated using propidium monoazide (PMA) in both workflows. Asterisks indicate statistically significant differences (* *p* < 0.05, ** *p* < 0.01) determined by the nonparametric Wilcoxon test.

The total populations (log_10_ copies/g) in the non-PMA control samples were 3.37 ± 0.09 in the on-site workflow and 3.37 ± 0.31 in the laboratory workflow (Figure 1). PMA treatment did not change total populations; however, the on-site workflow showed higher counts than the laboratory workflow in the PMA-treated samples.

Chao 1 richness in the non-PMA control samples was higher in the on-site than the laboratory workflow. In the PMA-treated samples, this pattern reversed: Chao 1 richness was higher in the laboratory than in the on-site workflow. The Shannon index was reduced in the laboratory workflow for both the non-PMA control and the PMA-treated samples. The difference between the workflows affected the evenness more than the PMA treatment.

In the non-PMA control samples, the delayed processing increased the abundance of Firmicutes relative to the on-site processing (Tables S1 and S2) and decreased the abundance of Proteobacteria. The non-PMA control in the laboratory workflow also showed higher abundance of Tenericutes and lower abundances of Bacteroidetes, Actinobacteria, Cyanobacteria, Verrucomicrobia, and OD1. After PMA treatment, the laboratory workflow samples remained enriched in Firmicutes and Tenericutes than the on-site workflow samples.

**Figure 2:**
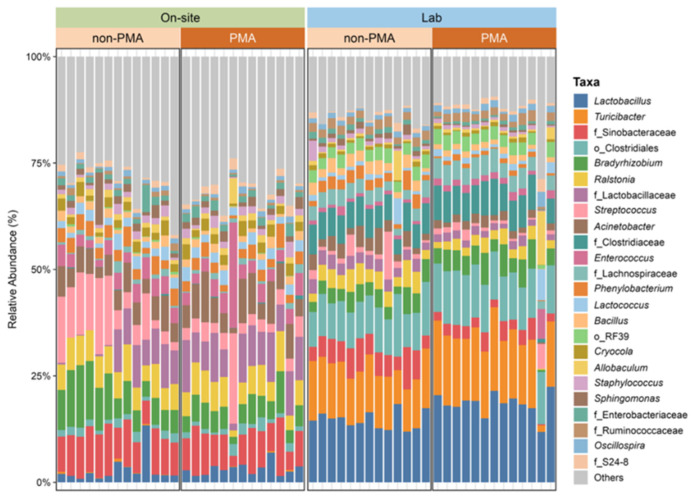
Relative abundances of the major genera (>1% in average abundance) in the bacterial microbiota of healthy cow’s milk subjected to immediate (freezing at the farm: on-site workflow) and delayed processing (freezing after cool transport: laboratory workflow). Viable and non-viable cells were discriminated using propidium monoazide (PMA) in both workflows.

In the non-PMA control samples, the abundances of *Lactobacillus*, *Turicibacter*, o_Clostridiale, and f_Clostridiaceae increased, and those of *Streptococcus*, f_Sinobacteraceae, *Bradyrhizobium*, *Ralstonia*, and *Acinetobacter* decreased in the laboratory compared with the on-site workflow (Figure 2, Tables S1 and S2). The abundances of *Staphylococcus*, f_Enterobacteriaceae, *Mycoplasma*, i.e., typical mastitis-related taxa, did not change, whereas those of *Streptococcus* and *Corynebacterium* decreased in the laboratory compared with the on-site workflow.

Although the relative abundances were changed, the profiles of prevalent families were retained both in the on-site and laboratory workflows after the PMA treatment (Figure 2, Tables S1 and S2). In the PMA-treated samples, the abundances of *Lactobacillus* and *Turicibacter* increased, and those of *Streptococcus* and f_Sinobacteraceae decreased in the laboratory compared with the on-site workflow. In contrast, the abundances of typical mastitis-related taxa were all affected by the PMA treatment in the laboratory workflow. The abundances of *Staphylococcus*, f_Enterobacteriaceae, *Mycoplasma*, *Streptococcus*, and *Corynebacterium* decreased in the laboratory compared with the on-site workflow. The PMA effects appeared more extensive in the laboratory workflow samples.

**Figure 3:**
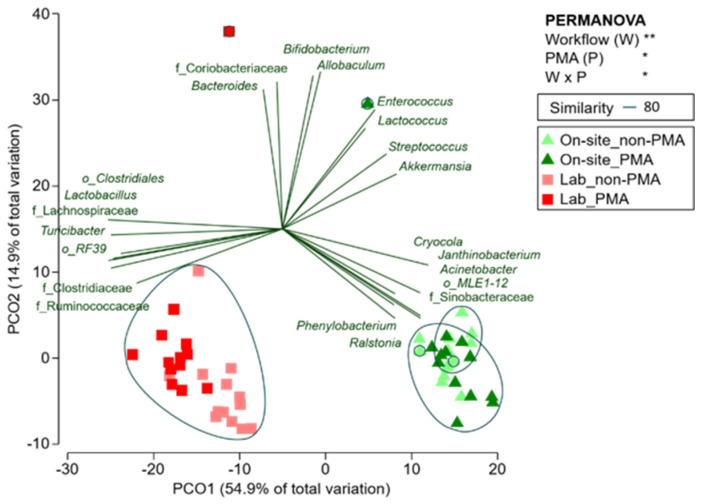
Principal coordinates plot characterizing the bacterial microbiota of healthy cow’s milk subjected to immediate (freezing at the farm: on-site workflow) and delayed processing (freezing after cool transport: laboratory workflow). Viable and non-viable cells were discriminated using propidium monoazide (PMA) in both workflows. Amplicon sequence variants with Pearson’s correlation > 0.7 are overlaid on the plot as vectors. Samples grouped at 80% similarity are denoted with green circles. Asterisks denote statistically significant PERMANOVA results (* *p* < 0.05, ** *p* < 0.01) for the effects of workflow (W), PMA treatment (P), and their interaction (W × P).

At the genus level, PERMANOVA showed significant effects of workflow (*p* < 0.01), PMA treatment (*p* < 0.05), and the interaction between workflow and PMA treatment (*p* < 0.05). In the PCoA, although the non-PMA control and PMA-treated samples largely overlapped in the laboratory workflow group (Figure 3). However, some of the PMA-treated samples showed a directional displacement from the other non-PMA control and PMA-treated samples in the on-site workflow group. The on-site workflow samples were characterized by f_Sinobacteraceae, *Acinetobacter*, *Ralstonia*, *Phenylobacterium*, *Janthinobacterium*, o_MLE1-12, and *Cryocola*, and the laboratory workflow samples were characterized by *Lactobacillus*, *Turicibacter*, o_Clostridiales, f_Clostridiaceae, f_Lactobacillaceae, o_RF39, and f_Ruminococcaceae.

### 3.2. Seasonal Variation Assessed in the On-Site Workflow

Sequencing of 72 milk samples generated 2,538,798 high-quality reads, ranging from 15,272 to 58,041 per sample. No month-specific imbalance in sequencing depth was evident, and the Wilcoxon test showed that non-chimeric read counts were similar between non-PMA control and PMA-treated samples in September, November, and January samples.

**Figure 4:**
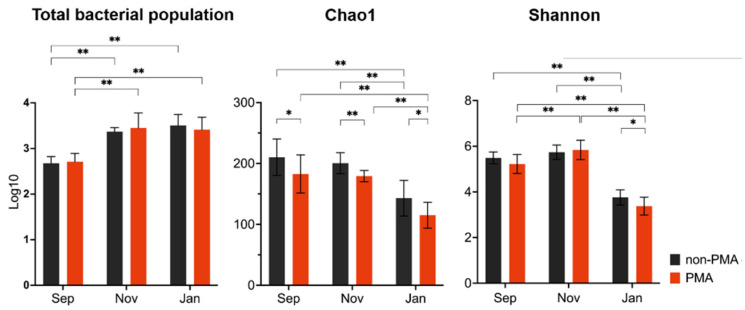
Total bacterial counts and alpha diversity indices of the bacterial microbiota in healthy cow’s milk collected in September 2023, November 2023, and January 2024. Samples were processed using the on-site workflow, with and without propidium monoazide (PMA) treatment to distinguish viable and non-viable cells. Asterisks indicate statistically significant differences (* *p* < 0.05, ** *p* < 0.01) determined by the nonparametric Wilcoxon test.

The bacterial populations (log_10_ copies/g) in the non-PMA control samples were 2.68 ± 0.15, 3.37 ± 0.09, and 3.51 ± 0.24 in September, November, and January, respectively (Figure 4). The number in September was lower than that in November and January, whereas the difference between November and January was not observed. The PMA-treated samples followed the same pattern: September differed from November and January. No difference was detected between non-PMA control and PMA-treated samples, regardless of season.

The Chao 1 richness of the non-PMA control samples was 210 ± 29.9, 201 ± 17.2, and 143 ± 29.2 in September, November, and January, respectively. No difference was seen between September and November, but the value in January was lower than that in September and November. The Shannon index followed the same pattern: the value was lower in January than in September and November. In the PMA-treated samples, the Chao 1 values were 183 ± 31.3, 179 ± 9.38, and 115 ± 21.3 in September, November, and January, respectively, with January differing from September and November. The Shannon index varied across the three seasons in the PMA-treated samples, increasing from September to November and then declining in January. Compared with the non-PMA control samples, Chao1 richness was lower in the PMA-treated samples regardless of season. The Shannon index was unaffected by PMA treatment in the September and November samples but was reduced in the January samples.

In the non-PMA control samples, the abundance of Proteobacteria increased from 34.4 ± 7.07% in September to 46.2 ± 7.47% in November, and 66.3 ± 5.95% in January (Tables S3 and S4). The abundance of Bacteroidetes increased from September to November and remained high in January. The abundances of Actinobacteria, Cyanobacteria, Verrucomicrobia, and OD1 peaked in November, whereas the highest abundance of Tenericutes was seen in September. The PMA-treated samples followed the same pattern across sampling seasons; however, in the September samples, the abundance of Proteobacteria was lower, and that of Firmicutes was higher compared with the non-PMA control samples.

**Figure 5:**
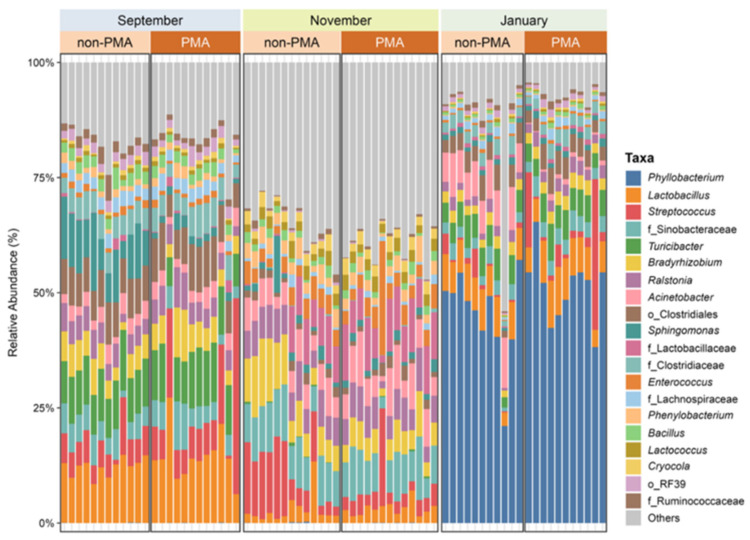
Relative abundances of the major genera (>1% average abundance) in the bacterial microbiota of healthy cow’s milk collected in September 2023, November 2023, and January 2024. Samples were processed using the on-site workflow, with and without propidium monoazide (PMA) treatment to distinguish viable and non-viable cells.

In this on-site workflow experiment, Lactobacillus, Turicibacter, and o_Clostridiales showed the highest abundance in September and the lowest abundance in November, and f_Sinobacteraceae and Ralstonia showed the highest abundance in November and the lowest abundance in January (Figure 5, Tables S3 and S4). The abundances of Streptococcus and Bradyrhizobium were similar in September and November, then decreased in January. Sphingomonas were the most abundant in September and then showed similar low values in November and January. Phyllobacterium indicated the opposite to Sphingomonas; the abundances were quite low in September and November and then showed as high as 45% in January. The abundance of Acinetobacter increased from September to January. The abundances of typical mastitis-related taxa other than Streptococcus, i.e., Corynebacterium, Staphylococcus, f_Enterobacteriaceae, and Mycoplasma, exhibited peaks in November, but the values (<2.0%) were much lower than those of prevalent taxa.

The pattern of seasonal variation and the abundance values were not affected by PMA treatment for *Lactobacillus*, *Turicibacter*, o_Clostridiales, f_Sinobacteraceae, *Bradyrhizobium*, and *Phyllobacterium* (Tables S3 and S4). Assessment of seasonal variation differed between the non-PMA control and PMA-treated samples for *Ralstonia*, *Streptococcus*, *Acinetobacter*, and *Sphingomonas*. After PMA treatment, *Streptococcus* showed the highest abundance in September and then decreased to similar values in November and January, and the difference between September and November became unclear for *Ralstonia*. The abundance in September samples was greatly reduced by PMA treatment, and the values in September and November became similar for *Sphingomonas*. The reduction of abundance by PMA treatment was extensive in January samples for *Acinetobacter*. January was the highest season in terms of abundance in the non-PMA control samples, but the lowest season in the PMA-treated samples.

**Figure 6:**
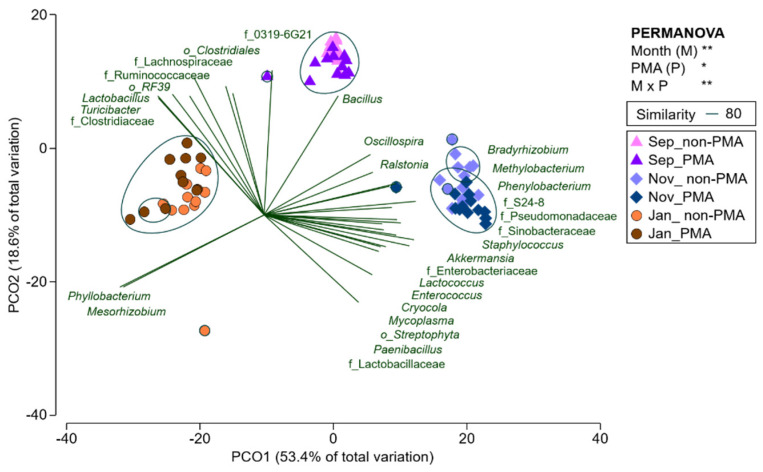
Principal coordinates plot characterizing the bacterial microbiota of healthy cow’s milk collected in September 2023, November 2023, and January 2024. Samples were processed using the on-site workflow, with and without propidium monoazide (PMA) treatment to distinguish viable and non-viable cells. Amplicon sequence variants with Pearson’s correlation >0.7 are overlaid on the plot as vectors. Samples grouped at 80% similarity are denoted with green circles. Asterisks denote statistically significant PERMANOVA results (* *p* < 0.05, ** *p* < 0.01) for the effects of workflow (W), PMA treatment (P), and their interaction (W × P).

PERMANOVA showed that the raw milk microbiota structure was affected by season at the genus level. The PMA treatment (*p* < 0.05) and the interaction between season and PMA treatment (*p* < 0.05) were also significant (Figure 6). The PCoA indicated that separation was driven mainly by season, whereas non-PMA control and PMA-treated samples largely overlapped despite the significant PMA effect. September and January samples were characterized by *Bacillus*, *Phyllobacterium*, and *Mesorhizobium*, respectively, while sharing *Lactobacillus*, *Turicibacter*, o_Clostridiales, f_Clostridiaceae, f_Lachnospiraceae, f_Ruminococcaceae, and o_RF39. November samples were characterized by many taxa, including f_Sinobacteriaceae, *Bradyrhizobium*, *Ralstonia*, *Phenylobacterium*, f_Enterobacteriaceae, *Enterococcus*, f_Lactobacillaceae, *Staphylococcus*, *Cryocola*, and *Mycoplasma*; however, *Streptococcus* and *Acinetobacter*, the top and fifth most abundant genera in the non-PMA control samples in November, were not included as the taxa that characterize the season.

## 4. Discussion

### 4.1. Sample Processing Workflow and Viability Assessment

The total bacterial population was not substantially affected by either workflow difference or PMA treatment; hence, bacteria detectable by DNA-based methods in raw cow’s milk can be regarded as mostly viable.

The total bacterial count remained largely unchanged by the workflow and PMA treatment. However, richness was altered by both the workflow and PMA treatment, and evenness was affected by the workflow; hence, delayed processing may influence the results of microbiota analysis. In the non-PMA samples, richness and evenness decreased in the laboratory workflow, suggesting that cold storage exerts a selective pressure on the microbiota. Likewise, the increase in richness observed in the PMA-treated samples in the laboratory workflow suggests selection for bacterial taxa adapted to cold storage conditions.

PMA treatment affected richness more than evenness, suggesting that the viability assessment may remove less abundant communities rather than alter the overall structure. Moreover, the different Chao 1 responses between workflows suggest that cold storage may alter membrane accessibility prior to dye treatment rather than simply preserve the original viable fraction [24,26,43]. The relative stability of the Shannon index across workflows suggests that prevalent taxa would remain even when richness shifts, which aligns with earlier PMA studies of raw milk microbiota [25,26].

The laboratory workflow increased the abundances of Firmicutes and Tenericutes and reduced the abundance of Proteobacteria relative to on-site processing, which aligns with the observation that PMA preferentially removes membrane-compromised Gram-negative taxa while retaining Gram-positive and spore-protected taxa [25,26,44].

Delayed processing shifted the microbiota profile from genera apparently linked to the environment, i.e., f_Sinobacteriaceae, *Bradyrhizobium*, *Ralstonia*, and *Acinetobacter*, to those linked to milk processing and the cow’s gut, i.e., *Lactobacillus*, *Turicibacter*, and f_Clostridiaceae. It has been demonstrated that soil-, plant-, and water-associated taxa can be detected in raw milk [3,45,46,47,48]. Whether these environmental taxa are vulnerable to low temperatures remains unclear, yet Xie et al. [3] detected *Bradyrhizobium* in raw milk during winter. *Lactobacillus* is among the dominant psychrophilic bacterial genera in raw milk, as reported by Xu et al. [49], along with *Pseudomonas*, *Stenotrophomonas*, *Sphingomonas*, and *Lactococcus*. *Turicibacter* has been shown to survive pasteurization [24]; thus, although it is not a spore-forming bacterium like *Bacillus* and *Clostridium*, the taxon would be more resistant to thermal changes than other taxa.

The abundances of *Staphylococcus*, *Corynebacterium*, *Mycoplasma*, and f_Enterobacteriaceae were not substantially different between the on-site and laboratory workflow; hence, although delayed processing due to cold storage from farm to laboratory may alter the community structure of raw milk microbiota [21,43,50], assessment of typical mastitis pathogens would not be endangered and could work further. However, the finding that the abundance of *Streptococcus* declined to approximately one-quarter due to delayed processing was an exception, as it suggests that laboratory-based assessment might underestimate its abundance in raw milk. Although many reports have shown a high relative abundance of *Streptococcus* in raw milk [8,9,14], higher levels might have been detected if the samples had been evaluated using the on-site workflow. Given that *Streptococcus* is associated with both mastitis and milk quality [9,47], this finding warrants further investigation. Regardless, the relative abundance of all typical mastitis-related taxa decreased following PMA treatment in the laboratory workflow; hence, it is unlikely that viable bacteria had been underestimated in previous laboratory workflow assessments.

*Pseudomonas* and *Acinetobacter*, typical taxa associated with low temperatures, were reduced by cold storage. Moreover, their viability was lower in the laboratory workflow than in the on-site workflow; hence, the results of this study did not support the hypothesis that *Pseudomonas* and *Acinetobacter* would increase with low-temperature storage. However, this inconsistency may indicate that cold enrichment in raw milk is selective and time-dependent. Raats et al. [21] reported that *Acinetobacter* became abundant, particularly after about 48 h of storage at 4 °C, and Rasolofo et al. [50] showed that at 4 °C, the relative abundance of *Acinetobacter* would decline when competing taxa, especially *Pseudomonas*, became dominant. Refrigerated storage may act as a selective enrichment process rather than a uniform increase of all psychrotrophs [51].

### 4.2. Seasonal Variation in the On-Site Workflow with Viability Assessment

The qPCR results indicated seasonal variation in bacterial load; bacterial populations were lowest in September and higher in November and January. Because PMA treatment did not alter qPCR counts across seasons, the finding in Experiment 1 that bacteria detectable by DNA-based methods in raw cow’s milk are mostly viable was confirmed. Although the view that total milk bacterial load varies with season has been demonstrated, the peak season differed among studies. Celano et al. [13] found higher mesophilic counts in winter than in summer; Kable et al. [10] reported higher bacterial numbers in spring than in fall; and Huck et al. [52] observed higher plate counts in spring and summer than in winter and autumn.

Distinctive declines in richness and evenness in January may indicate a restricted bacterial community enforced by cold environment. Similar to Experiment 1, effects of PMA treatment were seen more on the Chao 1 index than on the Shannon index, supporting the finding that viability assessment may remove less abundant communities rather than reconstruct the overall structure of milk microbiota [25,26].

Both non-PMA control and PMA-treated samples shifted from Firmicutes-rich profiles in September to Proteobacteria-dominant profiles in January. This phylum shift is consistent with reports of seasonal changes in environmental transfer into milk rather than the persistence of a stable phylum structure across sampling months [46,53,54].

Xie et al. [3] found that *Lactobacillus* and *Sphingomonas* were prevalent in raw milk microbiota during summer. A higher abundance of lactic acid bacterial taxa in summer was also reported by Celano et al. [13]. Although *Lactobacillus* was a taxon characterizing the September samples in this study, its relative abundance was also high in the coldest winter samples. The decrease in the abundances of *Turicibacter* and f_Clostridiaceae from September to November is consistent with results observed from July to November by Nguyen et al. [55]. However, the late-autumn decline in f_Clostridiaceae contrasts with the November peak in clostridial spores reported in raw milk by Komori et al. [56].

*Phyllobacterium*, a plant-associated taxon, became overwhelmingly dominant in January samples, which aligns with reports on winter microbiota of cow’s milk by Celano et al. [13]. *Phyllobacterium* was shown to increase in abundance during low-temperature storage of goat milk [23]. *Bradyrhizobium*, another plant-associated taxon, was prevalent across three seasons, peaking in November in this study. *Bradyrhizobium* has been identified as a core taxon in winter milk by Celano et al. [13] and as a biomarker in healthy cow milk in January samples by Xie et al. [3]. *Bacillus* and *Paenibacillus*, spore-forming genera, showed the lowest abundances in January samples, in contrast to Guo et al. [57], who reported that cold-tolerant *Bacillus* species became more abundant in the raw milk microbiota during winter. *Bacillus* and *Paenibacillus* are genera associated with feed, silage, feces, and teat contamination [58] and have been shown to persist from farm reservoirs in dairy processing systems [59,60,61].

*Streptococcus* was a prevalent taxon in September and November and then declined sharply in January samples in this study. This broadly aligns with the findings of Nguyen et al. [14], who reported a higher abundance of Streptococcaceae during the cool seasons from November to January, and with those of Yuan et al. [62], which indicated greater abundance of *Streptococcus* from September to December. The abundance of *Staphylococcus* peaked in November in our data set, despite Celano et al. [13], Nguyen et al. [14], and Kable et al. [10] reporting higher abundance in raw milk during summer. However, the abundances of typical mastitis-related taxa, except for *Streptococcus*, were low in this study, and the observed variations may be below levels that affect udder health.

### 4.3. Limitations of the Study and Future Directions

Although seasonal variation was evident, only three sampling times, conducted at two-month intervals, were included in this study. Furthermore, the study was conducted at a single farm; hence, the extent to which geographical differences and herd management practices may influence the results is unknown. Comparisons between the on-site and laboratory workflows were made only for the samples collected in November, and several cows were replaced during the study period. The effects of workflows and viability PCR need to be validated with more farms and milk samplings.

Regardless, our one-year monitoring of eight Jersey cows in the same region found that the milk microbiota of April, June, and December samples and that of February and October samples were separately grouped [15], indicating that seasonal variation may not be directly linked to climate-based classifications. Even as several cows were replaced, the ratio of primiparous to multiparous cows remained approximately 4:6 throughout the study, despite known differences in the milk microbiota between primiparous and multiparous cows [3]. Because the differences observed between the on-site and laboratory workflows were substantial, the finding that immediate freezing in the on-site workflow can yield a more accurate understanding of the milk microbiota is likely unchanged and should serve as a valuable addition for future studies.

Given the difficulty of performing PMA treatment on-site when sampling occurs across multiple farm visits, immediate freezing at the sampling site followed by transport to laboratories is a practical and feasible workflow. Since seasonal variations in raw milk microbiota are substantial, the value of existing knowledge and understanding obtained through laboratory workflows may remain unchanged. Schemes for raw milk microbiota monitoring need to continue improving to advance the diagnosis and prediction of dynamics of mastitis- and spoilage-related taxa.

## 5. Conclusions

The microbiota of raw milk from non-mastitic Holstein cow udders was evaluated differently depending on whether samples were frozen immediately at the collection site or transported to the laboratory under cool conditions. Opportunistic environmental taxa showed decreased abundance, while milk-processing and gut-associated taxa increased. Mastitis-related taxa remained largely unaffected. Viability PCR with propidium monoazide further modified the microbiota results, but the difference between viable and non-viable cells was minimal compared to the impact of delayed processing. Seasonal changes in raw milk microbiota were evident in samples processed immediately. Although the study has limitations, including a small number of milk samples and data from just one farm with a single management system, these results emphasize that sample handling from collection to analysis is crucial for accurate interpretation of udder milk microbiota, which is important for dairy cow health and product quality.

## Data Availability

The 16S rRNA gene sequencing data were deposited with links to BioProject accession number PRJDB42780 in the DDBJ BioProject database. Other datasets used and analyzed in this study are available from the corresponding author upon reasonable request.

## Supplementary Materials

The following supporting information can be downloaded at https://www.mdpi.com/article/10.3390/ani16152302/s1, Table S1: Relative abundances (%) at the phylum and genus levels of the microbiota in healthy cows’ milk, with statistical analyses clarifying differences between on-site and laboratory workflows, with and without PMA treatment; Table S2: Relative abundances (%) at the phylum and genus levels of the microbiota in healthy cow’s milk, with statistical analyses clarifying the effects of PMA treatment in the on-site and laboratory workflows; Table S3: Relative abundance (%) at the phylum and genus levels of the microbiota in healthy cow’s milk, with statistical analyses clarifying seasonal variations (September 2023, November 2023, and January 2024), with and without PMA treatment; Table S4: Relative abundances (%) at the phylum and genus levels of the microbiota in healthy cow’s milk, with statistical analyses clarifying the effects of PMA treatment in samples from September 2023, November 2023, and January 2024.
