# Supplementary material for: The Impact of Cold Storage and Seasonality on Raw Cow Milk Microbiota, Assessed by Conventional and Viability PCR
Source: Animals (Basel). 2026 Jul 24;16(15):2302. doi: 10.3390/ani16152302 (PMC13463561; doi:10.3390/ani16152302)
Supplement: Supplementary file 1 [file animals-16-02302-s001.zip › animals-4437761-supplementary.pdf]

Table S1. Relative abundances (%) at the phylum and genus levels of the microbiota in healthy cows' milk, with statistical analyses clarifying differences between on-site and laboratory workflows, with and without PMA treatment

	Non-PMA control			PMA		
**Phylum/Genus**	**On-site**	**Lab**	**Test**	**On-site**	**Lab**	**Test**
Actinobacteria	4.21 ± 1.06	1.64 ± 0.46	**	5.28 ± 1.72	2.31 ± 3.47	**
Cryocola	2.14 ± 0.78	0.78 ± 0.27	**	2.37 ± 0.79	0.65 ± 0.31	**
Bifidobacterium	0.51 ± 0.32	0.20 ± 0.23	*	1.23 ± 1.88	0.95 ± 2.53	**
Corynebacterium	0.58 ± 0.33	0.20 ± 0.28	**	0.35 ± 0.14	0.12 ± 0.06	**
f_Coriobacteriaceae	0.07 ± 0.11	0.01 ± 0.02	NS	0.12 ± 0.33	0.32 ± 0.97	NS
Bacteroidetes	3.67 ± 0.88	2.39 ± 1.41	**	4.84 ± 1.73	3.25 ± 1.92	**
f_S24-8	1.02 ± 0.45	0.70 ± 0.55	NS	1.38 ± 0.73	0.96 ± 0.67	NS
Bacteroides	0.27 ± 0.28	0.39 ± 0.64	NS	0.58 ± 1.08	0.55 ± 1.12	NS
Prevotella	0.44 ± 0.35	0.20 ± 0.22	*	0.48 ± 0.26	0.43 ± 0.23	NS
Parabacteroides	0.33 ± 0.29	0.20 ± 0.12	NS	0.43 ± 0.31	0.14 ± 0.13	*
o_Bacteroidales	0.14 ± 0.16	0.17 ± 0.23	NS	0.28 ± 0.29	0.43 ± 0.29	NS
Flavobacterium	0.27 ± 0.15	0.17 ± 0.12	NS	0.39 ± 0.32	0.14 ± 0.12	NS
Pedobacter	0.20 ± 0.19	0.05 ± 0.07	*	0.28 ± 0.25	0.04 ± 0.07	*
f_Rikenellaceae	0.10 ± 0.13	0.09 ± 0.13	NS	0.18 ± 0.32	0.15 ± 0.33	NS
Chryseobacterium	0.16 ± 0.16	0.04 ± 0.06	*	0.12 ± 0.14	0.08 ± 0.14	NS
Cloacibacterium	0.12 ± 0.15	0.06 ± 0.06	NS	0.02 ± 0.04	0.02 ± 0.04	NS
Cyanobacteria	2.99 ± 2.38	1.15 ± 0.61	**	1.95 ± 0.85	0.60 ± 0.49	**
o_MLE1-12	1.35 ± 0.48	0.84 ± 0.47	*	1.22 ± 0.68	0.33 ± 0.20	**
o_Streptophyta	0.58 ± 0.48	0.22 ± 0.22	*	0.45 ± 0.38	0.22 ± 0.38	*
Nostoc	0.64 ± 2.29	0.00 ± 0.00	NS	0.00 ± 0.00	0.00 ± 0.00	NS
Chroococcidiopsis	0.03 ± 0.09	0.00 ± 0.00	NS	0.00 ± 0.01	0.00 ± 0.00	NS
Firmicutes	36.9 ± 6.08	64.6 ± 5.37	**	38.2 ± 10.27	73.0 ± 4.10	**
Lactobacillus	2.91 ± 3.32	14.6 ± 2.05	**	3.11 ± 1.48	18.4 ± 2.73	**
Turicibacter	0.17 ± 0.23	12.2 ± 1.74	**	0.07 ± 0.16	15.0 ± 4.30	**
o_Clostridiales	1.61 ± 0.58	9.60 ± 1.99	**	1.54 ± 0.85	11.6 ± 0.81	**
f_Lactobacillaceae	4.78 ± 4.53	2.86 ± 0.54	NS	8.70 ± 3.05	1.63 ± 0.61	**
Streptococcus	9.26 ± 6.03	2.20 ± 2.97	**	4.57 ± 5.08	1.41 ± 1.46	**
f_Clostridiaceae	0.08 ± 0.12	6.22 ± 0.98	**	0.15 ± 0.25	6.78 ± 1.96	**
Enterococcus	3.51 ± 1.10	0.99 ± 0.38	**	5.14 ± 5.97	1.99 ± 1.55	**
f_Lachnospiraceae	1.00 ± 0.67	3.19 ± 0.74	**	0.87 ± 0.55	4.16 ± 0.98	**
Lactococcus	2.22 ± 0.84	1.29 ± 1.46	**	2.43 ± 0.59	1.63 ± 1.77	**
Bacillus	1.55 ± 0.63	2.44 ± 0.82	**	1.49 ± 0.70	1.30 ± 0.44	NS
Allobaculum	1.15 ± 0.43	0.96 ± 1.82	**	1.63 ± 1.36	1.66 ± 3.33	*
Staphylococcus	1.40 ± 0.65	1.27 ± 1.08	NS	1.74 ± 0.86	0.74 ± 0.26	**
f_Ruminococcaceae	0.73 ± 0.54	2.07 ± 0.26	**	0.40 ± 0.18	1.80 ± 0.59	**
Oscillospira	0.97 ± 0.29	1.04 ± 0.22	NS	0.88 ± 0.41	1.41 ± 0.75	*
Paenibacillus	1.01 ± 1.04	0.72 ± 0.25	NS	1.67 ± 0.68	0.22 ± 0.12	**
Ruminococcus	0.36 ± 0.25	0.48 ± 0.18	NS	0.52 ± 0.22	0.64 ± 0.20	NS
Clostridium	0.68 ± 0.43	0.17 ± 0.10	**	0.62 ± 0.28	0.37 ± 0.22	*
Aerococcus	0.66 ± 1.71	0.27 ± 0.61	NS	0.06 ± 0.11	0.10 ± 0.08	*
Coprococcus	0.23 ± 0.32	0.06 ± 0.06	NS	0.14 ± 0.18	0.14 ± 0.18	NS
f_Peptostreptococcaceae	0.14 ± 0.12	0.05 ± 0.06	*	0.18 ± 0.21	0.03 ± 0.04	NS
Geobacillus	0.00 ± 0.00	0.00 ± 0.00	NS	0.11 ± 0.38	0.00 ± 0.00	NS
Anaerovibrio	0.01 ± 0.02	0.00 ± 0.00	NS	0.00 ± 0.00	0.00 ± 0.02	NS
Proteobacteria	46.2 ± 7.47	24.8 ± 6.03	**	41.5 ± 11.2	16.3 ± 5.63	**
f_Sinobacteraceae	9.16 ± 1.95	5.55 ± 1.98	**	8.20 ± 2.98	2.88 ± 1.34	**
Bradyrhizobium	8.45 ± 3.82	3.78 ± 1.29	**	5.14 ± 2.00	4.73 ± 2.03	NS
Ralstonia	6.51 ± 1.31	3.86 ± 1.34	**	6.02 ± 2.05	2.36 ± 0.95	**

Acinetobacter	5.37 ± 1.58	3.00 ± 1.48	**	6.86 ± 3.14	1.71 ± 0.63	**
Phenylobacterium	3.15 ± 0.96	2.46 ± 0.98	NS	2.61 ± 1.18	0.85 ± 0.39	**
Sphingomonas	2.25 ± 3.08	1.06 ± 0.56	NS	1.50 ± 0.70	0.29 ± 0.16	**
f_Enterobacteriaceae	1.64 ± 1.46	1.08 ± 0.30	NS	1.66 ± 0.72	0.63 ± 0.29	**
Burkholderia	1.44 ± 1.35	0.74 ± 0.28	*	1.06 ± 0.38	0.50 ± 0.19	**
f_Pseudomonadaceae	1.41 ± 1.16	0.59 ± 0.30	**	1.24 ± 0.64	0.50 ± 0.29	**
Pseudomonas	0.91 ± 0.46	0.33 ± 0.31	**	1.14 ± 0.86	0.41 ± 0.22	**
Janthinobacterium	0.79 ± 0.34	0.40 ± 0.23	**	1.03 ± 0.49	0.28 ± 0.14	**
f_Aeromonadaceae	0.40 ± 0.40	0.08 ± 0.13	**	0.53 ± 0.47	0.09 ± 0.08	**
Methylobacterium	0.41 ± 0.40	0.14 ± 0.09	**	0.33 ± 0.22	0.07 ± 0.08	**
f_Neisseriaceae	0.33 ± 0.37	0.14 ± 0.17	NS	0.32 ± 0.24	0.12 ± 0.10	*
f_Comamonadaceae	0.26 ± 0.22	0.12 ± 0.15	NS	0.28 ± 0.39	0.10 ± 0.09	NS
f_Methylobacteriaceae	0.27 ± 0.46	0.08 ± 0.05	NS	0.24 ± 0.20	0.03 ± 0.04	**
f_Oxalobacteraceae	0.09 ± 0.08	0.06 ± 0.13	NS	0.23 ± 0.32	0.01 ± 0.03	**
Phyllobacterium	0.05 ± 0.09	0.02 ± 0.04	NS	0.03 ± 0.04	0.02 ± 0.03	NS
f_Sphingomonadaceae	0.09 ± 0.34	0.00 ± 0.01	NS	0.00 ± 0.02	0.00 ± 0.00	NS
f_0319-6G21	0.03 ± 0.08	0.02 ± 0.04	NS	0.01 ± 0.03	0.00 ± 0.00	NS
Massilia	0.04 ± 0.13	0.00 ± 0.00	NS	0.00 ± 0.00	0.00 ± 0.00	NS
Mesorhizobium	0.00 ± 0.00	0.00 ± 0.00	NS	0.01 ± 0.03	0.00 ± 0.00	NS
Tenericutes	0.69 ± 0.33	3.24 ± 0.38	**	0.64 ± 0.43	3.09 ± 0.65	**
o_RF39	0.26 ± 0.22	2.89 ± 0.33	**	0.07 ± 0.12	2.94 ± 0.71	**
Mycoplasma	0.41 ± 0.22	0.33 ± 0.19	NS	0.56 ± 0.36	0.13 ± 0.08	**
Verrucomicrobia	0.94 ± 0.31	0.38 ± 0.15	**	0.76 ± 0.37	0.44 ± 0.33	*
Akkermansia	0.86 ± 0.29	0.34 ± 0.13	**	0.73 ± 0.36	0.39 ± 0.34	**
OD1	0.38 ± 0.21	0.11 ± 0.08	**	0.41 ± 0.34	0.06 ± 0.05	**
Others	3.47 ± 2.41	1.38 ± 0.57	**	5.64 ± 4.45	0.60 ± 0.43	**

Phyla and genera with a relative abundance of >1% in at least one sample are indicated. **: $p < 0.01$, *: $p < 0.05$, NS: not significant.

Table S2. Relative abundances (%) at the phylum and genus levels of the microbiota in healthy cow's milk, with statistical analyses clarifying the effects of PMA treatment in the on-site and laboratory workflows

**Phylum/Genus**	**On-site**			**Lab**		
	**Non-PMA control**	**PMA**	**Test**	**Non-PMA control**	**PMA**	**Test**
Actinobacteria	4.21 ± 1.06	5.28 ± 1.72	NS	1.64 ± 0.46	2.31 ± 3.47	NS
Cryocola	2.14 ± 0.78	2.37 ± 0.79	NS	0.78 ± 0.27	0.65 ± 0.31	NS
Bifidobacterium	0.51 ± 0.32	1.23 ± 1.88	NS	0.20 ± 0.23	0.95 ± 2.53	NS
Corynebacterium	0.58 ± 0.33	0.35 ± 0.14	NS	0.20 ± 0.28	0.12 ± 0.06	NS
f_Coriobacteriaceae	0.07 ± 0.11	0.12 ± 0.33	NS	0.01 ± 0.02	0.32 ± 0.97	*
Bacteroidetes	3.67 ± 0.88	4.84 ± 1.73	NS	2.39 ± 1.41	3.25 ± 1.92	NS
f_S24-8	1.02 ± 0.45	1.38 ± 0.73	NS	0.70 ± 0.55	0.96 ± 0.67	NS
Bacteroides	0.27 ± 0.28	0.58 ± 1.08	NS	0.39 ± 0.64	0.55 ± 1.12	NS
Prevotella	0.44 ± 0.35	0.48 ± 0.26	NS	0.20 ± 0.22	0.43 ± 0.23	*
Parabacteroides	0.33 ± 0.29	0.43 ± 0.31	NS	0.20 ± 0.12	0.14 ± 0.13	NS
o_Bacteroidales	0.14 ± 0.16	0.28 ± 0.29	NS	0.17 ± 0.23	0.43 ± 0.29	*
Flavobacterium	0.27 ± 0.15	0.39 ± 0.32	NS	0.17 ± 0.12	0.14 ± 0.12	NS
Pedobacter	0.20 ± 0.19	0.28 ± 0.25	NS	0.05 ± 0.07	0.04 ± 0.07	NS
f_Rikenellaceae	0.10 ± 0.13	0.18 ± 0.32	NS	0.09 ± 0.13	0.15 ± 0.33	NS
Chryseobacterium	0.16 ± 0.16	0.12 ± 0.14	NS	0.04 ± 0.06	0.08 ± 0.14	NS
Cloacibacterium	0.12 ± 0.15	0.02 ± 0.04	NS	0.06 ± 0.06	0.02 ± 0.04	NS
Cyanobacteria	2.99 ± 2.38	1.95 ± 0.85	NS	1.15 ± 0.61	0.60 ± 0.49	*
o_MLE1-12	1.35 ± 0.48	1.22 ± 0.68	NS	0.84 ± 0.47	0.33 ± 0.20	**
o_Streptophyta	0.58 ± 0.48	0.45 ± 0.38	NS	0.22 ± 0.22	0.22 ± 0.38	NS
Nostoc	0.64 ± 2.29	0.00 ± 0.00	NS	0.00 ± 0.00	0.00 ± 0.00	NS
Chroococcidiopsis	0.03 ± 0.09	0.00 ± 0.01	NS	0.00 ± 0.00	0.00 ± 0.00	NS
Firmicutes	36.9 ± 6.08	38.2 ± 10.3	NS	64.6 ± 5.37	73.0 ± 4.10	**
Lactobacillus	2.91 ± 3.32	3.11 ± 1.48	NS	14.62 ± 2.05	18.40 ± 2.73	**
Turicibacter	0.17 ± 0.23	0.07 ± 0.16	NS	12.17 ± 1.74	14.98 ± 4.30	**
o_Clostridiales	1.61 ± 0.58	1.54 ± 0.85	NS	9.60 ± 1.99	11.55 ± 0.81	**
f_Lactobacillaceae	4.78 ± 4.53	8.70 ± 3.05	*	2.86 ± 0.54	1.63 ± 0.61	**
Streptococcus	9.26 ± 6.03	4.57 ± 5.08	NS	2.20 ± 2.97	1.41 ± 1.46	NS
f_Clostridiaceae	0.08 ± 0.12	0.15 ± 0.25	NS	6.22 ± 0.98	6.78 ± 1.96	NS
Enterococcus	3.51 ± 1.10	5.14 ± 5.97	NS	0.99 ± 0.38	1.99 ± 1.55	**
f_Lachnospiraceae	1.00 ± 0.67	0.87 ± 0.55	NS	3.19 ± 0.74	4.16 ± 0.98	*
Lactococcus	2.22 ± 0.84	2.43 ± 0.59	NS	1.29 ± 1.46	1.63 ± 1.77	NS
Bacillus	1.55 ± 0.63	1.49 ± 0.70	NS	2.44 ± 0.82	1.30 ± 0.44	**
Allobaculum	1.15 ± 0.43	1.63 ± 1.36	NS	0.96 ± 1.82	1.66 ± 3.33	NS
Staphylococcus	1.40 ± 0.65	1.74 ± 0.86	NS	1.27 ± 1.08	0.74 ± 0.26	*
f_Ruminococcaceae	0.73 ± 0.54	0.40 ± 0.18	NS	2.07 ± 0.26	1.80 ± 0.59	NS
Oscillospira	0.97 ± 0.29	0.88 ± 0.41	NS	1.04 ± 0.22	1.41 ± 0.75	NS
Paenibacillus	1.01 ± 1.04	1.67 ± 0.68	NS	0.72 ± 0.25	0.22 ± 0.12	**
Ruminococcus	0.36 ± 0.25	0.52 ± 0.22	NS	0.48 ± 0.18	0.64 ± 0.20	NS
Clostridium	0.68 ± 0.43	0.62 ± 0.28	NS	0.17 ± 0.10	0.37 ± 0.22	**
Aerococcus	0.66 ± 1.71	0.06 ± 0.11	*	0.27 ± 0.61	0.10 ± 0.08	NS
Coprococcus	0.23 ± 0.32	0.14 ± 0.18	NS	0.06 ± 0.06	0.14 ± 0.18	NS

f_Peptostreptococcaceae	0.14 ± 0.12	0.18 ± 0.21	NS	0.05 ± 0.06	0.03 ± 0.04	NS
Geobacillus	0.00 ± 0.00	0.11 ± 0.38	NS	0.00 ± 0.00	0.00 ± 0.00	NS
Anaerovibrio	0.01 ± 0.02	0.00 ± 0.00	NS	0.00 ± 0.00	0.00 ± 0.02	NS
Proteobacteria	46.2 ± 7.47	41.5 ± 11.2	NS	24.8 ± 6.03	16.3 ± 5.63	**
f_Sinobacteraceae	9.16 ± 1.95	8.20 ± 2.98	NS	5.55 ± 1.98	2.88 ± 1.34	**
Bradyrhizobium	8.45 ± 3.82	5.14 ± 2.00	*	3.78 ± 1.29	4.73 ± 2.03	NS
Ralstonia	6.51 ± 1.31	6.02 ± 2.05	NS	3.86 ± 1.34	2.36 ± 0.95	**
Acinetobacter	5.37 ± 1.58	6.86 ± 3.14	NS	3.00 ± 1.48	1.71 ± 0.63	*
Phenylobacterium	3.15 ± 0.96	2.61 ± 1.18	NS	2.46 ± 0.98	0.85 ± 0.39	**
Sphingomonas	2.25 ± 3.08	1.50 ± 0.70	NS	1.06 ± 0.56	0.29 ± 0.16	**
f_Enterobacteriaceae	1.64 ± 1.46	1.66 ± 0.72	NS	1.08 ± 0.30	0.63 ± 0.29	**
Burkholderia	1.44 ± 1.35	1.06 ± 0.38	NS	0.74 ± 0.28	0.50 ± 0.19	*
f_Pseudomonadaceae	1.41 ± 1.16	1.24 ± 0.64	NS	0.59 ± 0.30	0.50 ± 0.29	NS
Pseudomonas	0.91 ± 0.46	1.14 ± 0.86	NS	0.33 ± 0.31	0.41 ± 0.22	NS
Janthinobacterium	0.79 ± 0.34	1.03 ± 0.49	NS	0.40 ± 0.23	0.28 ± 0.14	NS
f_Aeromonadaceae	0.40 ± 0.40	0.53 ± 0.47	NS	0.08 ± 0.13	0.09 ± 0.08	NS
Methylobacterium	0.41 ± 0.40	0.33 ± 0.22	NS	0.14 ± 0.09	0.07 ± 0.08	*
f_Neisseriaceae	0.33 ± 0.37	0.32 ± 0.24	NS	0.14 ± 0.17	0.12 ± 0.10	NS
f_Comamonadaceae	0.26 ± 0.22	0.28 ± 0.39	NS	0.12 ± 0.15	0.10 ± 0.09	NS
f_Methylobacteriaceae	0.27 ± 0.46	0.24 ± 0.20	NS	0.08 ± 0.05	0.03 ± 0.04	*
f_Oxalobacteraceae	0.09 ± 0.08	0.23 ± 0.32	NS	0.06 ± 0.13	0.01 ± 0.03	NS
Phyllobacterium	0.05 ± 0.09	0.03 ± 0.04	NS	0.02 ± 0.04	0.02 ± 0.03	NS
f_Sphingomonadaceae	0.09 ± 0.34	0.00 ± 0.02	NS	0.00 ± 0.01	0.00 ± 0.00	NS
f_0319-6G21	0.03 ± 0.08	0.01 ± 0.03	NS	0.02 ± 0.04	0.00 ± 0.00	*
Massilia	0.04 ± 0.13	0.00 ± 0.00	NS	0.00 ± 0.00	0.00 ± 0.00	NS
Mesorhizobium	0.00 ± 0.00	0.01 ± 0.03	NS	0.00 ± 0.00	0.00 ± 0.00	NS
Tenericutes	0.69 ± 0.33	0.64 ± 0.43	NS	3.24 ± 0.38	3.09 ± 0.65	NS
o_RF39	0.26 ± 0.22	0.07 ± 0.12	**	2.89 ± 0.33	2.94 ± 0.71	NS
Mycoplasma	0.41 ± 0.22	0.56 ± 0.36	NS	0.33 ± 0.19	0.13 ± 0.08	**
Verrucomicrobia	0.94 ± 0.31	0.76 ± 0.37	NS	0.38 ± 0.15	0.44 ± 0.33	NS
Akkermansia	0.86 ± 0.29	0.73 ± 0.36	NS	0.34 ± 0.13	0.39 ± 0.34	NS
OD1	0.38 ± 0.21	0.41 ± 0.34	NS	0.11 ± 0.08	0.06 ± 0.05	NS
Others	3.47 ± 2.41	5.64 ± 4.45	NS	1.38 ± 0.57	0.60 ± 0.43	**

Phyla and genera with a relative abundance of >1% in at least one sample are indicated. **: $p < 0.01$, *: $p < 0.05$, NS: not significant.

Table S3. Relative abundance (%) at the phylum and genus levels of the microbiota in healthy cow's milk, with statistical analyses clarifying seasonal variations (September 2023, November 2023, and January 2024) , with and without PMA treatment

Phylum/ Genus	Non-PMA control						PMA					
	Sep	Nov	Jan	Sep vs Nov	Nov vs Jan	Sep vs Jan	Sep	Nov	Jan	Sep vs Nov	Nov vs Jan	Sep vs Jan
Actinobacteria	1.57 ± 0.43	4.21 ± 1.06	0.68 ± 0.40	**	**	**	1.70 ± 0.69	5.28 ± 1.72	0.61 ± 0.27	**	**	**
Cryocola	0.68 ± 0.30	2.14 ± 0.78	0.27 ± 0.14	**	**	**	0.82 ± 0.31	2.37 ± 0.79	0.38 ± 0.18	**	**	**
Bifidobacterium	0.26 ± 0.34	0.51 ± 0.32	0.07 ± 0.06	*	**	*	0.36 ± 0.75	1.23 ± 1.88	0.12 ± 0.21	**	**	NS
Corynebacterium	0.18 ± 0.11	0.58 ± 0.33	0.23 ± 0.32	**	**	NS	0.14 ± 0.10	0.35 ± 0.14	0.04 ± 0.05	**	**	*
f_Coriobacteriaceae	0.09 ± 0.09	0.07 ± 0.11	0.01 ± 0.02	NS	NS	**	0.07 ± 0.06	0.12 ± 0.33	0.01 ± 0.02	NS	NS	**
Bacteroidetes	2.34 ± 0.76	3.67 ± 0.88	4.05 ± 9.28	**	**	*	1.89 ± 0.61	4.84 ± 1.73	0.90 ± 0.41	**	**	**
f_S24-8	0.71 ± 0.30	1.02 ± 0.45	0.18 ± 0.10	NS	**	**	0.53 ± 0.28	1.38 ± 0.73	0.25 ± 0.18	**	**	*
Bacteroides	0.20 ± 0.13	0.27 ± 0.28	0.06 ± 0.04	NS	NS	*	0.20 ± 0.14	0.58 ± 1.08	0.06 ± 0.04	NS	**	**
Prevotella	0.25 ± 0.16	0.44 ± 0.35	0.10 ± 0.05	NS	**	*	0.24 ± 0.20	0.48 ± 0.26	0.13 ± 0.10	*	**	NS
Parabacteroides	0.11 ± 0.10	0.33 ± 0.29	0.09 ± 0.06	NS	*	NS	0.17 ± 0.17	0.43 ± 0.31	0.10 ± 0.05	*	**	NS
o_Bacteroidales	0.19 ± 0.21	0.14 ± 0.16	0.19 ± 0.16	NS	NS	NS	0.12 ± 0.11	0.28 ± 0.29	0.14 ± 0.12	NS	NS	NS
Flavobacterium	0.11 ± 0.12	0.27 ± 0.15	2.66 ± 7.86	**	NS	NS	0.11 ± 0.10	0.39 ± 0.32	0.03 ± 0.03	*	**	NS
Pedobacter	0.03 ± 0.03	0.20 ± 0.19	0.38 ± 1.24	*	NS	NS	0.07 ± 0.08	0.28 ± 0.25	0.02 ± 0.03	*	**	NS
f_Rikenellaceae	0.15 ± 0.14	0.10 ± 0.13	0.03 ± 0.06	NS	NS	**	0.07 ± 0.07	0.18 ± 0.32	0.03 ± 0.04	NS	NS	NS
Chryseobacterium	0.06 ± 0.06	0.16 ± 0.16	0.15 ± 0.35	NS	NS	NS	0.05 ± 0.06	0.12 ± 0.14	0.03 ± 0.04	NS	*	NS
Cloacibacterium	0.04 ± 0.05	0.12 ± 0.15	0.10 ± 0.30	NS	NS	NS	0.02 ± 0.03	0.02 ± 0.04	0.01 ± 0.01	NS	NS	NS
Cyanobacteria	1.03 ± 0.70	2.99 ± 2.38	0.35 ± 0.15	**	**	**	0.78 ± 0.30	1.95 ± 0.85	0.40 ± 0.14	**	**	**
o_MLE1-12	0.66 ± 0.38	1.35 ± 0.48	0.29 ± 0.17	**	**	*	0.67 ± 0.27	1.22 ± 0.68	0.37 ± 0.13	*	**	**
o_Streptophyta	0.15 ± 0.22	0.58 ± 0.48	0.04 ± 0.04	**	**	*	0.04 ± 0.06	0.45 ± 0.38	0.02 ± 0.02	**	**	NS
Nostoc	0.00 ± 0.00	0.64 ± 2.29	0.00 ± 0.00	NS	NS	NS	0.00 ± 0.00	0.00 ± 0.00	0.00 ± 0.00	NS	NS	NS
Chroococcidiopsis	0.18 ± 0.61	0.03 ± 0.09	0.00 ± 0.00	NS	NS	NS	0.01 ± 0.02	0.00 ± 0.01	0.00 ± 0.00	NS	NS	NS
Firmicutes	57.3 ± 6.03	36.9 ± 6.08	26.8 ± 6.76	**	**	**	64.2 ± 8.01	38.2 ± 10.3	31.8 ± 7.95	**	NS	**
Lactobacillus	12.2 ± 1.91	2.91 ± 3.32	7.20 ± 1.62	**	**	**	14.6 ± 5.40	3.11 ± 1.48	7.52 ± 2.18	**	**	**
Turicibacter	10.1 ± 1.29	0.17 ± 0.23	5.24 ± 1.40	**	**	**	10.6 ± 2.21	0.07 ± 0.16	5.35 ± 1.37	**	**	**
o_Clostridiales	7.80 ± 0.70	1.61 ± 0.58	3.99 ± 1.22	**	**	**	8.24 ± 1.97	1.54 ± 0.85	3.97 ± 1.15	**	**	**
f_Lactobacillaceae	0.46 ± 0.74	4.78 ± 4.53	0.64 ± 0.24	*	NS	*	0.57 ± 0.85	8.70 ± 3.05	0.78 ± 0.28	**	**	*
Streptococcus	5.83 ± 2.62	9.26 ± 6.03	1.52 ± 1.97	NS	**	**	11.0 ± 11.2	4.57 ± 5.08	5.81 ± 10.0	**	NS	*
f_Clostridiaceae	4.83 ± 0.69	0.08 ± 0.12	2.64 ± 0.76	**	**	**	4.99 ± 1.46	0.15 ± 0.25	2.65 ± 0.59	**	**	**
Enterococcus	1.15 ± 0.43	3.51 ± 1.10	0.47 ± 0.16	**	**	**	1.02 ± 0.41	5.14 ± 5.97	0.50 ± 0.24	**	**	**
f_Lachnospiraceae	2.74 ± 0.39	1.00 ± 0.67	1.38 ± 0.55	**	NS	**	2.64 ± 0.62	0.87 ± 0.55	1.36 ± 0.59	**	*	**
Lactococcus	0.83 ± 0.19	2.22 ± 0.84	0.28 ± 0.18	**	**	**	0.80 ± 0.21	2.43 ± 0.59	0.37 ± 0.14	**	**	**
Bacillus	2.69 ± 0.79	1.55 ± 0.63	0.60 ± 0.16	**	**	**	2.29 ± 0.57	1.49 ± 0.70	0.75 ± 0.27	**	**	**
Allobaculum	0.70 ± 0.40	1.15 ± 0.43	0.18 ± 0.08	*	**	**	1.75 ± 3.26	1.63 ± 1.36	0.39 ± 0.56	NS	**	**
Staphylococcus	0.73 ± 0.21	1.40 ± 0.65	0.21 ± 0.19	**	**	**	0.66 ± 0.38	1.74 ± 0.86	0.19 ± 0.17	**	**	**
f_Ruminococcaceae	2.26 ± 1.27	0.73 ± 0.54	0.91 ± 0.40	**	NS	**	1.47 ± 0.29	0.40 ± 0.18	0.78 ± 0.39	**	**	**
Oscillospira	0.98 ± 0.29	0.97 ± 0.29	0.37 ± 0.13	NS	**	**	0.84 ± 0.21	0.88 ± 0.41	0.32 ± 0.14	NS	**	**
Paenibacillus	0.16 ± 0.14	1.01 ± 1.04	0.10 ± 0.06	NS	*	NS	0.24 ± 0.27	1.67 ± 0.68	0.15 ± 0.12	**	**	NS

Ruminococcus	0.38 ± 0.15	0.36 ± 0.25	0.12 ± 0.08	NS	*	**	0.57 ± 0.30	0.52 ± 0.22	0.17 ± 0.09	NS	**	**
Clostridium	0.53 ± 0.98	0.68 ± 0.43	0.07 ± 0.08	**	**	**	0.22 ± 0.14	0.62 ± 0.28	0.12 ± 0.10	**	**	NS
Aerococcus	0.42 ± 0.56	0.66 ± 1.71	0.09 ± 0.09	NS	NS	*	0.10 ± 0.08	0.06 ± 0.11	0.03 ± 0.06	*	NS	*
Coprococcus	0.07 ± 0.08	0.23 ± 0.32	0.02 ± 0.02	NS	NS	NS	0.08 ± 0.08	0.14 ± 0.18	0.04 ± 0.09	NS	NS	NS
f_Peptostreptococcaceae	0.10 ± 0.15	0.14 ± 0.12	0.01 ± 0.01	NS	*	NS	0.16 ± 0.43	0.18 ± 0.21	0.02 ± 0.04	NS	*	NS
Geobacillus	0.00 ± 0.00	0.00 ± 0.00	0.00 ± 0.00	NS	NS	NS	0.02 ± 0.03	0.11 ± 0.38	0.00 ± 0.00	NS	NS	NS
Anaerovibrio	0.15 ± 0.50	0.01 ± 0.02	0.00 ± 0.00	NS	NS	NS	0.01 ± 0.02	0.00 ± 0.00	0.00 ± 0.00	NS	NS	NS
Proteobacteria	34.4 ± 7.07	46.2 ± 7.47	66.3 ± 5.95	**	**	**	28.1 ± 8.41	41.5 ± 11.15	64.7 ± 8.13	**	**	**
f_Sinobacteraceae	5.03 ± 1.60	9.16 ± 1.95	2.56 ± 0.86	**	**	**	5.24 ± 2.61	8.20 ± 2.98	3.35 ± 0.66	*	**	*
Bradyrhizobium	5.26 ± 1.76	8.45 ± 3.82	2.30 ± 0.50	NS	**	**	5.79 ± 2.70	5.14 ± 2.00	2.75 ± 0.40	NS	**	**
Ralstonia	4.61 ± 1.26	6.51 ± 1.31	1.91 ± 0.72	**	**	**	5.03 ± 1.86	6.02 ± 2.05	2.63 ± 0.51	NS	**	**
Acinetobacter	2.98 ± 1.01	5.37 ± 1.58	7.85 ± 1.57	**	**	**	2.95 ± 1.06	6.86 ± 3.14	0.77 ± 0.39	**	**	**
Phenylobacterium	1.45 ± 0.47	3.15 ± 0.96	0.21 ± 0.06	**	**	**	1.51 ± 0.81	2.61 ± 1.18	0.24 ± 0.06	**	**	**
Sphingomonas	9.91 ± 3.60	2.25 ± 3.08	1.16 ± 0.26	**	NS	**	1.78 ± 0.73	1.50 ± 0.70	1.26 ± 0.24	NS	NS	*
f_Enterobacteriaceae	0.63 ± 0.20	1.64 ± 1.46	0.34 ± 0.09	**	**	**	0.68 ± 0.17	1.66 ± 0.72	0.22 ± 0.11	**	**	**
Burkholderia	0.86 ± 0.26	1.44 ± 1.35	0.38 ± 0.15	NS	**	**	0.85 ± 0.34	1.06 ± 0.38	0.51 ± 0.15	NS	**	**
f_Pseudomonadaceae	0.50 ± 0.21	1.41 ± 1.16	0.31 ± 0.16	**	**	*	0.59 ± 0.30	1.24 ± 0.64	0.27 ± 0.18	**	**	**
Pseudomonas	0.42 ± 0.32	0.91 ± 0.46	0.61 ± 1.28	**	**	NS	0.39 ± 0.21	1.14 ± 0.86	0.15 ± 0.15	**	**	**
Janthinobacterium	0.31 ± 0.18	0.79 ± 0.34	0.68 ± 1.47	**	*	NS	0.31 ± 0.18	1.03 ± 0.49	0.10 ± 0.09	**	**	**
f_Aeromonadaceae	0.09 ± 0.12	0.40 ± 0.40	0.21 ± 0.42	*	NS	NS	0.06 ± 0.08	0.53 ± 0.47	0.02 ± 0.04	**	**	NS
Methylobacterium	0.22 ± 0.11	0.41 ± 0.40	0.02 ± 0.03	NS	**	**	0.15 ± 0.05	0.33 ± 0.22	0.02 ± 0.04	*	**	**
f_Neisseriaceae	0.01 ± 0.04	0.33 ± 0.37	0.02 ± 0.03	**	**	NS	0.08 ± 0.13	0.32 ± 0.24	0.02 ± 0.02	**	**	NS
f_Comamonadaceae	0.08 ± 0.08	0.26 ± 0.22	0.79 ± 2.27	*	NS	NS	0.13 ± 0.12	0.28 ± 0.39	0.03 ± 0.05	NS	*	*
f_Methylobacteriaceae	0.03 ± 0.03	0.27 ± 0.46	0.00 ± 0.00	*	**	*	0.05 ± 0.06	0.24 ± 0.20	0.00 ± 0.01	**	**	NS
f_Oxalobacteraceae	0.08 ± 0.11	0.09 ± 0.08	0.02 ± 0.04	NS	*	NS	0.30 ± 1.02	0.23 ± 0.32	0.00 ± 0.00	*	**	NS
Phyllobacterium	0.02 ± 0.03	0.05 ± 0.09	45.3 ± 9.74	NS	**	**	0.02 ± 0.04	0.03 ± 0.04	51.1 ± 7.30	NS	**	**
f_Sphingomonadaceae	0.00 ± 0.00	0.09 ± 0.34	0.00 ± 0.00	NS	NS	NS	0.00 ± 0.01	0.00 ± 0.02	0.00 ± 0.00	NS	NS	NS
f_0319-6G21	0.60 ± 0.24	0.03 ± 0.08	0.00 ± 0.00	**	NS	**	0.76 ± 0.44	0.01 ± 0.03	0.00 ± 0.00	**	NS	**
Massilia	0.02 ± 0.06	0.04 ± 0.13	0.00 ± 0.00	NS	NS	NS	0.34 ± 1.13	0.00 ± 0.00	0.00 ± 0.00	*	NS	*
Mesorhizobium	0.00 ± 0.00	0.00 ± 0.00	1.21 ± 0.36	NS	**	**	0.00 ± 0.00	0.01 ± 0.03	1.10 ± 0.30	NS	**	**
Tenericutes	2.29 ± 0.44	0.69 ± 0.33	1.07 ± 0.32	**	*	**	2.27 ± 0.96	0.64 ± 0.43	1.16 ± 0.43	**	*	**
o_RF39	2.14 ± 0.43	0.26 ± 0.22	1.01 ± 0.29	**	**	**	2.15 ± 0.94	0.07 ± 0.12	1.12 ± 0.44	**	**	**
Mycoplasma	0.13 ± 0.09	0.41 ± 0.22	0.05 ± 0.03	**	**	*	0.11 ± 0.10	0.56 ± 0.36	0.03 ± 0.03	**	**	NS
Verrucomicrobia	0.38 ± 0.28	0.94 ± 0.31	0.20 ± 0.14	**	**	NS	0.40 ± 0.23	0.76 ± 0.37	0.19 ± 0.12	**	**	**
Akkermansia	0.36 ± 0.27	0.86 ± 0.29	0.19 ± 0.13	**	**	NS	0.38 ± 0.24	0.73 ± 0.36	0.18 ± 0.11	*	**	**
OD1	0.03 ± 0.04	0.38 ± 0.21	0.02 ± 0.02	**	**	NS	0.07 ± 0.07	0.41 ± 0.34	0.03 ± 0.03	**	**	*
Others	0.36 ± 0.14	3.47 ± 2.41	0.34 ± 0.18	**	**	NS	0.40 ± 0.26	5.64 ± 4.45	0.12 ± 0.10	**	**	**

Phylum and genera with a relative abundance of >1% in at least one sample are indicated. **: $p < 0.01$, *: $p < 0.05$, NS: not significant.

Table S4. Relative abundances (%) at the phylum and genus levels of the microbiota in healthy cow's milk, with statistical analyses clarifying the effects of PMA treatment in samples from September 2023, November 2023, and January 2024

	September			November			January		
**Phylum/Genus**	**Non-PMA control**	**PMA**	**Test**	**Non-PMA control**	**PMA**	**Test**	**Non-PMA control**	**PMA**	**Test**
Actinobacteria	1.57 ± 0.43	1.70 ± 0.69	NS	4.21 ± 1.06	5.28 ± 1.72	NS	0.68 ± 0.40	0.61 ± 0.27	NS
Cryocola	0.68 ± 0.30	0.82 ± 0.31	NS	2.14 ± 0.78	2.37 ± 0.79	NS	0.27 ± 0.14	0.38 ± 0.18	NS
Bifidobacterium	0.26 ± 0.34	0.36 ± 0.75	NS	0.51 ± 0.32	1.23 ± 1.88	NS	0.07 ± 0.06	0.12 ± 0.21	NS
Corynebacterium	0.18 ± 0.11	0.14 ± 0.10	NS	0.58 ± 0.33	0.35 ± 0.14	NS	0.23 ± 0.32	0.04 ± 0.05	*
f_Coriobacteriaceae	0.09 ± 0.09	0.07 ± 0.06	NS	0.07 ± 0.11	0.12 ± 0.33	NS	0.01 ± 0.02	0.01 ± 0.02	NS
Bacteroidetes	2.34 ± 0.76	1.89 ± 0.61	NS	3.67 ± 0.88	4.84 ± 1.73	NS	4.05 ± 9.28	0.90 ± 0.41	NS
f_S24-8	0.71 ± 0.30	0.53 ± 0.28	NS	1.02 ± 0.45	1.38 ± 0.73	NS	0.18 ± 0.10	0.25 ± 0.18	NS
Bacteroides	0.20 ± 0.13	0.20 ± 0.14	NS	0.27 ± 0.28	0.58 ± 1.08	NS	0.06 ± 0.04	0.06 ± 0.04	NS
Prevotella	0.25 ± 0.16	0.24 ± 0.20	NS	0.44 ± 0.35	0.48 ± 0.26	NS	0.10 ± 0.05	0.13 ± 0.10	NS
Parabacteroides	0.11 ± 0.10	0.17 ± 0.17	NS	0.33 ± 0.29	0.43 ± 0.31	NS	0.09 ± 0.06	0.10 ± 0.05	NS
o_Bacteroidales	0.19 ± 0.21	0.12 ± 0.11	NS	0.14 ± 0.16	0.28 ± 0.29	NS	0.19 ± 0.16	0.14 ± 0.12	NS
Flavobacterium	0.11 ± 0.12	0.11 ± 0.10	NS	0.27 ± 0.15	0.39 ± 0.32	NS	2.66 ± 7.86	0.03 ± 0.03	*
Pedobacter	0.03 ± 0.03	0.07 ± 0.08	NS	0.20 ± 0.19	0.28 ± 0.25	NS	0.38 ± 1.24	0.02 ± 0.03	NS
f_Rikenellaceae	0.15 ± 0.14	0.07 ± 0.07	NS	0.10 ± 0.13	0.18 ± 0.32	NS	0.03 ± 0.06	0.03 ± 0.04	NS
Chryseobacterium	0.06 ± 0.06	0.05 ± 0.06	NS	0.16 ± 0.16	0.12 ± 0.14	NS	0.15 ± 0.35	0.03 ± 0.04	NS
Cloacibacterium	0.04 ± 0.05	0.02 ± 0.03	NS	0.12 ± 0.15	0.02 ± 0.04	NS	0.10 ± 0.30	0.01 ± 0.01	NS
Cyanobacteria	1.03 ± 0.70	0.78 ± 0.30	NS	2.99 ± 2.38	1.95 ± 0.85	NS	0.35 ± 0.15	0.40 ± 0.14	NS
o_MLE1-12	0.66 ± 0.38	0.67 ± 0.27	NS	1.35 ± 0.48	1.22 ± 0.68	NS	0.29 ± 0.17	0.37 ± 0.13	NS
o_Streptophyta	0.15 ± 0.22	0.04 ± 0.06	*	0.58 ± 0.48	0.45 ± 0.38	NS	0.04 ± 0.04	0.02 ± 0.02	NS
Nostoc	0.00 ± 0.00	0.00 ± 0.00	NS	0.64 ± 2.29	0.00 ± 0.00	NS	0.00 ± 0.00	0.00 ± 0.00	NS
Chroococcidiopsis	0.18 ± 0.61	0.01 ± 0.02	NS	0.03 ± 0.09	0.00 ± 0.01	NS	0.00 ± 0.00	0.00 ± 0.00	NS
Firmicutes	57.3 ± 6.03	64.2 ± 8.01	*	36.9 ± 6.08	38.2 ± 10.3	NS	26.8 ± 6.76	31.8 ± 7.95	NS
Lactobacillus	12.2 ± 1.91	14.6 ± 5.40	NS	2.91 ± 3.32	3.11 ± 1.48	NS	7.20 ± 1.62	7.52 ± 2.18	NS
Turicibacter	10.1 ± 1.29	10.6 ± 2.21	NS	0.17 ± 0.23	0.07 ± 0.16	NS	5.24 ± 1.40	5.35 ± 1.37	NS
o_Clostridiales	7.80 ± 0.70	8.24 ± 1.97	NS	1.61 ± 0.58	1.54 ± 0.85	NS	3.99 ± 1.22	3.97 ± 1.15	NS
f_Lactobacillaceae	0.46 ± 0.74	0.57 ± 0.85	NS	4.78 ± 4.53	8.70 ± 3.05	*	0.64 ± 0.24	0.78 ± 0.28	NS
Streptococcus	5.83 ± 2.62	11.0 ± 11.2	*	9.26 ± 6.03	4.57 ± 5.08	NS	1.52 ± 1.97	5.81 ± 10.0	NS
f_Clostridiaceae	4.83 ± 0.69	4.99 ± 1.46	NS	0.08 ± 0.12	0.15 ± 0.25	NS	2.64 ± 0.76	2.65 ± 0.59	NS
Enterococcus	1.15 ± 0.43	1.02 ± 0.41	NS	3.51 ± 1.10	5.14 ± 5.97	NS	0.47 ± 0.16	0.50 ± 0.24	NS
f_Lachnospiraceae	2.74 ± 0.39	2.64 ± 0.62	NS	1.00 ± 0.67	0.87 ± 0.55	NS	1.38 ± 0.55	1.36 ± 0.59	NS
Lactococcus	0.83 ± 0.19	0.80 ± 0.21	NS	2.22 ± 0.84	2.43 ± 0.59	NS	0.28 ± 0.18	0.37 ± 0.14	NS
Bacillus	2.69 ± 0.79	2.29 ± 0.57	NS	1.55 ± 0.63	1.49 ± 0.70	NS	0.60 ± 0.16	0.75 ± 0.27	NS
Allobaculum	0.70 ± 0.40	1.75 ± 3.26	NS	1.15 ± 0.43	1.63 ± 1.36	NS	0.18 ± 0.08	0.39 ± 0.56	NS
Staphylococcus	0.73 ± 0.21	0.66 ± 0.38	NS	1.40 ± 0.65	1.74 ± 0.86	NS	0.21 ± 0.19	0.19 ± 0.17	NS
f_Ruminococcaceae	2.26 ± 1.27	1.47 ± 0.29	*	0.73 ± 0.54	0.40 ± 0.18	NS	0.91 ± 0.40	0.78 ± 0.39	NS
Oscillospira	0.98 ± 0.29	0.84 ± 0.21	NS	0.97 ± 0.29	0.88 ± 0.41	NS	0.37 ± 0.13	0.32 ± 0.14	NS
Paenibacillus	0.16 ± 0.14	0.24 ± 0.27	NS	1.01 ± 1.04	1.67 ± 0.68	NS	0.10 ± 0.06	0.15 ± 0.12	NS
Ruminococcus	0.38 ± 0.15	0.57 ± 0.30	NS	0.36 ± 0.25	0.52 ± 0.22	NS	0.12 ± 0.08	0.17 ± 0.09	NS

Clostridium	0.53 ± 0.98	0.22 ± 0.14	NS	0.68 ± 0.43	0.62 ± 0.28	NS	0.07 ± 0.08	0.12 ± 0.10	NS
Aerococcus	0.42 ± 0.56	0.10 ± 0.08	*	0.66 ± 1.71	0.06 ± 0.11	*	0.09 ± 0.09	0.03 ± 0.06	NS
Coprococcus	0.07 ± 0.08	0.08 ± 0.08	NS	0.23 ± 0.32	0.14 ± 0.18	NS	0.02 ± 0.02	0.04 ± 0.09	NS
f_Peptostreptococcaceae	0.10 ± 0.15	0.16 ± 0.43	NS	0.14 ± 0.12	0.18 ± 0.21	NS	0.01 ± 0.01	0.02 ± 0.04	NS
Geobacillus	0.00 ± 0.00	0.02 ± 0.03	NS	0.00 ± 0.00	0.11 ± 0.38	NS	0.00 ± 0.00	0.00 ± 0.00	NS
Anaerovibrio	0.15 ± 0.50	0.01 ± 0.02	NS	0.01 ± 0.02	0.00 ± 0.00	NS	0.00 ± 0.00	0.00 ± 0.00	NS
Proteobacteria	34.4 ± 7.07	28.1 ± 8.41	*	46.2 ± 7.47	41.5 ± 11.2	NS	66.3 ± 5.95	64.7 ± 8.13	NS
f_Sinobacteraceae	5.03 ± 1.60	5.24 ± 2.61	NS	9.16 ± 1.95	8.20 ± 2.98	NS	2.56 ± 0.86	3.35 ± 0.66	*
Bradyrhizobium	5.26 ± 1.76	5.79 ± 2.70	NS	8.45 ± 3.82	5.14 ± 2.00	*	2.30 ± 0.50	2.75 ± 0.40	NS
Ralstonia	4.61 ± 1.26	5.03 ± 1.86	NS	6.51 ± 1.31	6.02 ± 2.05	NS	1.91 ± 0.72	2.63 ± 0.51	*
Acinetobacter	2.98 ± 1.01	2.95 ± 1.06	NS	5.37 ± 1.58	6.86 ± 3.14	NS	7.85 ± 1.57	0.77 ± 0.39	**
Phenylobacterium	1.45 ± 0.47	1.51 ± 0.81	NS	3.15 ± 0.96	2.61 ± 1.18	NS	0.21 ± 0.06	0.24 ± 0.06	NS
Sphingomonas	9.91 ± 3.60	1.78 ± 0.73	**	2.25 ± 3.08	1.50 ± 0.70	NS	1.16 ± 0.26	1.26 ± 0.24	NS
f_Enterobacteriaceae	0.63 ± 0.20	0.68 ± 0.17	NS	1.64 ± 1.46	1.66 ± 0.72	NS	0.34 ± 0.09	0.22 ± 0.11	**
Burkholderia	0.86 ± 0.26	0.85 ± 0.34	NS	1.44 ± 1.35	1.06 ± 0.38	NS	0.38 ± 0.15	0.51 ± 0.15	NS
f_Pseudomonadaceae	0.50 ± 0.21	0.59 ± 0.30	NS	1.41 ± 1.16	1.24 ± 0.64	NS	0.31 ± 0.16	0.27 ± 0.18	NS
Pseudomonas	0.42 ± 0.32	0.39 ± 0.21	NS	0.91 ± 0.46	1.14 ± 0.86	NS	0.61 ± 1.28	0.15 ± 0.15	NS
Janthinobacterium	0.31 ± 0.18	0.31 ± 0.18	NS	0.79 ± 0.34	1.03 ± 0.49	NS	0.68 ± 1.47	0.10 ± 0.09	NS
f_Aeromonadaceae	0.09 ± 0.12	0.06 ± 0.08	NS	0.40 ± 0.40	0.53 ± 0.47	NS	0.21 ± 0.42	0.02 ± 0.04	*
Methylobacterium	0.22 ± 0.11	0.15 ± 0.05	NS	0.41 ± 0.40	0.33 ± 0.22	NS	0.02 ± 0.03	0.02 ± 0.04	NS
f_Neisseriaceae	0.01 ± 0.04	0.08 ± 0.13	NS	0.33 ± 0.37	0.32 ± 0.24	NS	0.02 ± 0.03	0.02 ± 0.02	NS
f_Comamonadaceae	0.08 ± 0.08	0.13 ± 0.12	NS	0.26 ± 0.22	0.28 ± 0.39	NS	0.79 ± 2.27	0.03 ± 0.05	*
f_Methylobacteriaceae	0.03 ± 0.03	0.05 ± 0.06	NS	0.27 ± 0.46	0.24 ± 0.20	NS	0.00 ± 0.00	0.00 ± 0.01	NS
f_Oxalobacteraceae	0.08 ± 0.11	0.30 ± 1.02	NS	0.09 ± 0.08	0.23 ± 0.32	NS	0.02 ± 0.04	0.00 ± 0.00	NS
Phyllobacterium	0.02 ± 0.03	0.02 ± 0.04	NS	0.05 ± 0.09	0.03 ± 0.04	NS	45.3 ± 9.74	51.1 ± 7.30	NS
f_Sphingomonadaceae	0.00 ± 0.00	0.00 ± 0.01	NS	0.09 ± 0.34	0.00 ± 0.02	NS	0.00 ± 0.00	0.00 ± 0.00	NS
f_0319-6G21	0.60 ± 0.24	0.76 ± 0.44	NS	0.03 ± 0.08	0.01 ± 0.03	NS	0.00 ± 0.00	0.00 ± 0.00	NS
Massilia	0.02 ± 0.06	0.34 ± 1.13	NS	0.04 ± 0.13	0.00 ± 0.00	NS	0.00 ± 0.00	0.00 ± 0.00	NS
Mesorhizobium	0.00 ± 0.00	0.00 ± 0.00	NS	0.00 ± 0.00	0.01 ± 0.03	NS	1.21 ± 0.36	1.10 ± 0.30	NS
Tenericutes	2.29 ± 0.44	2.27 ± 0.96	NS	0.69 ± 0.33	0.64 ± 0.43	NS	1.07 ± 0.32	1.16 ± 0.43	NS
o_RF39	2.14 ± 0.43	2.15 ± 0.94	NS	0.26 ± 0.22	0.07 ± 0.12	**	1.01 ± 0.29	1.12 ± 0.44	NS
Mycoplasma	0.13 ± 0.09	0.11 ± 0.10	NS	0.41 ± 0.22	0.56 ± 0.36	NS	0.05 ± 0.03	0.03 ± 0.03	NS
Verrucomicrobia	0.38 ± 0.28	0.40 ± 0.23	NS	0.94 ± 0.31	0.76 ± 0.37	NS	0.20 ± 0.14	0.19 ± 0.12	NS
Akkermansia	0.36 ± 0.27	0.38 ± 0.24	NS	0.86 ± 0.29	0.73 ± 0.36	NS	0.19 ± 0.13	0.18 ± 0.11	NS
OD1	0.03 ± 0.04	0.07 ± 0.07	*	0.38 ± 0.21	0.41 ± 0.34	NS	0.02 ± 0.02	0.03 ± 0.03	NS
Others	0.36 ± 0.14	0.40 ± 0.26	NS	3.47 ± 2.41	5.64 ± 4.45	NS	0.34 ± 0.18	0.12 ± 0.10	**

Phyla and genera with a relative abundance of >1% in at least one sample are indicated. **: $p < 0.01$, *: $p < 0.05$, NS: not significant.
